# Hybrid Surgical Guidance in Urologic Robotic Oncological Surgery

**DOI:** 10.3390/jcm14176128

**Published:** 2025-08-29

**Authors:** Gijs H. KleinJan, Erik J. van Gennep, Arnoud W. Postema, Fijs W. B. van Leeuwen, Tessa Buckle

**Affiliations:** 1 Department of Urology, Leiden University Medical Center, 2333 ZA Leiden, The Netherlands; 2 Interventional Molecular Imaging Laboratory, Leiden University Medical Center, 2333 ZC Leiden, The Netherlands

**Keywords:** hybrid tracers, robot assisted surgery, urology, prostate cancer, kidney cancer, bladder cancer, image guided surgery

## Abstract

Urologic oncological surgery increasingly makes use of robotic systems to realize precise and minimally invasive resections, convent to shorter hospital stays and faster recovery times. The dexterity gains enabled through procedures such as robot-assisted (RA) prostatectomy have helped realize significant advancements in recent years. Complementing these effects via the used of hybrid tracers that illuminate surgical targets, i.e., cancerous tissue, has helped advance the surgical decision making via enhanced visualization. A well-known example is Indocyanine green (ICG)-Technetium-99m (^99m^Tc)-nanocolloid, a hybrid extension of the radiopharmaceutical ^99m^Tc-nanocolloid. These hybrid tracers provide a direct link between preoperative imaging roadmaps and intraoperative target identification, and improve efficiency, accuracy, and confidence of the urologist in procedures such as sentinel lymph node biopsy (SLNB). Receptor-targeted hybrid tracer analogues, for e.g., prostate specific membrane antigen (PSMA), are also being explored as an extension of the ongoing efforts that use radiotracers such as ^99m^Tc-PSMA-I&S. Together, these efforts jointly pave the way for novel techniques in intraoperative lesion localization in other urological malignancies. This narrative review discusses the potential use of hybrid tracers in robotic oncological urology, including different imaging techniques and their applications for tumor localization for prostate, bladder, and kidney cancer.

## 1. Introduction

Robotic-urology has made significant progress in recent years, with robot-assisted (RA) prostatectomy being a notable example [1]. Robotic-urology aims to improve surgical outcomes by incorporating dexterous, precise, and minimally invasive techniques along with detailed intraoperative (molecular) imaging information that guides surgical decision making [2]. Hereby, the latter provides the surgeon with improved perception of different tissues located within the surgical wound [3]. The use of radiopharmaceutical tracers that accumulate in surgical targets has created a key advancement in non-invasive diagnostics, e.g., sentinel lymph node biopsy (SLNB), single-photon emission tomography (SPECT) and prostate specific membrane antigen (PSMA), and positron emission tomography (PET). These approaches help provide surgical ‘roadmaps’ for the localization of primary cancers as well as micro- and macro-metastatic lymphatic disease, especially when combined with anatomical imaging (e.g., computed tomography (CT) imaging) [4]. One of today’s key challenges is to translate these preoperative insights into intraoperative precision guidance. This process, however, is complicated by the requirement for different imaging-labels and thus tracers to support preoperative (radiotracer), intraoperative (radiotracer and/or fluorescent tracer) guidance.

A surgeons ability to implement intraoperative imaging in robotics has been facilitated by: (1) the integration of fluorescence laparoscopy into robotic systems (e.g., Firefly fluorescence imaging, Intuitive Surgical and vLimeLite Fluorescence Imaging System, CMR Surgical, [5]), (2) The commercial availability of tethered gamma-detectors, so-called drop-in gamma probes (e.g., Crystal Photonics and Sensei Lightpoint, [6,7]), and (3) The increasing availability of alternative modalities such as a drop-in beta probe [8], Cerenkov luminescence imaging [9] and robot-assisted SPECT [10]. All these technologies independently support fluorescence or radioguidance, but can help to achieve best-of-both-worlds scenarios when combined with the use of hybrid tracers [11]. Hereby, the latter are essentially chemical modifications of radiotracers that set the current standard for preoperative imaging and subsequent intraoperative beta- or gamma-tracing [4,11].

**Figure 1:**
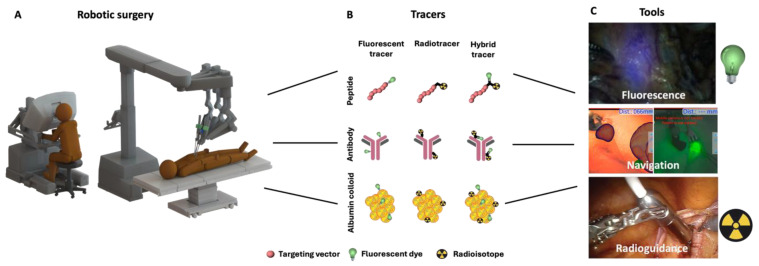
Image-guidance in robotic surgery. (**A**) Robot surgery, (**B**) available tracer designs with varying targeting moieties and imaging labels that allow image guidance, and (**C**) tools for intraoperative guidance. Fluorescence image obtained from KleinJan et al., Eur Urol 2014 [14], Navigation images obtained from KleinJan et al., JNM 2016 [15], and radioguidance (gamma Drop in probe) image obtained from van Gennep et al., EJNMMI 2025 [16].

Ongoing efforts indicate that complementation of radio- with fluorescence guidance enhances a surgeon’s ability to locate and remove cancerous tissue while preserving healthy tissue as much as possible [11,12,13]. In parallel, the introduction of hybrid tracers also paves the way for the clinical translation of new medical devices that help further enhance the detection accuracy (Figure 1). This narrative review elaborates on the potential use of hybrid tracers in robotic oncological urology. This includes different imaging techniques and their applications for tumor localization for prostate, bladder, and kidney cancer.

## 2. Methods

A narrative review was conducted utilizing databases such as PubMed, EMBASE, and the Cochrane Database of Systematic Reviews and grey literature. Relevant keywords were selected from the 2023 Medical Subject Headings (MeSH) online database. The search strategy encompassed terms such as ‘radiosurgery’, ‘fluorescence’, ‘hybrid imaging’, ‘hybrid imaging agents’, ‘robotic surgery’, ‘robot-assisted surgery’, ‘prostate cancer’, ‘bladder cancer’, ‘renal cancer’, and ‘kidney cancer’. The narrative review focused primarily on articles published from January 2010 onwards to capture the most recent advancements in the field, while also considering studies published between 2000 and 2025. In addition to database searches, manual searches were conducted through reference tracing and exploration of grey literature, namely conference abstracts and unpublished data from possible relevant theses.

## 3. Results

### 3.1. Tracer Designs

#### 3.1.1. Radiotracers

Preoperative imaging prior to urological surgery is generally based on CT, PET, and SPECT imaging [11,17]. Studies in SLNB have shown the additional value of combining nuclear imaging with CT to elevate the imaging approach from 2D lymphoscintigraphy to 3D SPECT/CT. A similar combination is used for PSMA-targeted approaches using either SPECT/CT or PET/CT [18]. The created overlay of images provides both functional and anatomical information and serves as a roadmap for surgical planning and initial intraoperative guidance towards the region of interest within the surgical field [17].

The most applied tracers for radioguided surgery contain the gamma emitter Technetium-99m (^99m^Tc) as a radiolabel, which is compatible with SPECT imaging. With its short half-life of about 6 h, it is ideal for use in medical imaging as it minimizes radiation exposure to patients. Primary examples of such tracers for use in robotic surgery are ^99m^Tc-nanocolloid [11] for use in SLNB and ^99m^Tc-PSMA-I&S for tumor-targeted approaches in salvage surgery [12,13,18,19]. Another frequently applied gamma isotope is ^111^Indium (half-life of ^111^In is approximately 2.8 days) with tracer examples being ^111^In-capromab pendetide (Prostascint) [20] and multiple different ^111^In-labelled (peptide based) PSMA tracers, as described in a systematic review by Berrens et al. [12]. Gamma tracers are generally applied the day of or the day before surgery and allow intraoperative gamma tracing with either a gamma camera or, in the case of robotic surgery, with a gamma drop-in probe [21].

Tracers labeled with beta-emitting isotopes such as Gallium-68 (Ga^68^) (half-life 68 min) are compatible with PET imaging [22]. While these tracers are primarily used for diagnostic imaging [23], specialized beta-probes have also been successfully used for intraoperative detection [24]. Alternatively, the beta-radiation can be detected using specialized Cerenkov radiation detectors [25,26]. Cerenkov luminescence is light (peak emission < 250nm) that is emitted when high-energy charged particles, such as radioactive electrons and positrons, travel through a medium at a speed faster than the speed of light in that medium. This provides a secondary optical readout for radioactive decay that can be detected using highly sensitive optical cameras. Oderda et al. used this technology to conduct an intraoperative assessment of surgical margins during prostatectomy [27].

#### 3.1.2. Fluorescent Dyes

The use of fluorescent dyes for intraoperative visualization of surgical targets during robotic surgery has become more popular in the last two decades. Most frequently, fluorescence is used for the identification of vascular structures and verification of adequate perfusion using ICG (excitation 800 nm/emission 820 nm, [28,29]). This technique has been applied, for e.g., identification of the renal arteries in case of (super) selective clamping in partial nephrectomy and the visualization of the vasculature and tumor in partial nephrectomies based on negative contrast [29,30,31]. In SLNB, ICG is applied as a lymphangiography tracer, visualizing lymphatic vessels and the sentinel node (SN) [32]. A different fluorescent dye is fluorescein (excitation 494 nm/emission 521 nm), which is excreted by the kidney and can therefore be used to visualize the ureters [28,33], but can also be used for the evaluation of damage to the ureters or urine leakage [34]. For both dyes, imaging is facilitated by specialized and often integrated fluorescence cameras (e.g., Firefly, Da Vinci, Intuitive Surgical, [10,32]).

#### 3.1.3. Tracer Design That Facilitates Combined Preoperative and Intraoperative Guidance

Hybrid tracer designs combine a radiolabel with a fluorescent dye component, and in the case of targeted tracers, also a targeting vector/moiety (Figure 1B) [35]. Hybrid SLNB tracers are generally based on colloids that consist of human serum albumin aggerates (such as nanocolloid, nanoscan, and nanotop [3,36,37]) and are applied to facilitate indirect detection of micrometastasis by visualizing the most likely nodal landing sites for tumor cells that are taken from the primary cancer by lymphatic fluid.

Hybrid SLNB tracers have been compared to reference standards such as ^99m^Tc-nanocolloid [38], ^99m^Tc-nanoscan [34], Blue dye [37], ICG [10], or combinations thereof. In all cases, the hybrid SLNB tracers maintained the diagnostic accuracy of the radiocolloid, while providing accurate optical intraoperative SLN identification. This approach compensates for the limited visualization rates of blue dye (only 22–78% [39]) and oversampling caused when solely using ICG [10]. Studies that have compared outcomes all indicate that the hybrid SLNB tracers yield more favorable results than combinations of individual components [39,40].

Examples of PSMA-targeted hybrid agents are summarized in the review by Jiao et al. [9]. The only available hybrid tracer for clinical studies for tumor targeting in kidney cancer is moment ^111^In-girentuximab-IRDye800CW [41].

### 3.2. Devices for Intraoperative Detection

Intraoperative identification requires supplemental detection modalities that are complementary to the preoperative imaging method used, for instance, radiotracers and gamma probes for radioguidance and fluorescent/hybrid tracers for fluorescence guidance with a fluorescence laparoscope. In addition, these signals can also be exploited for navigation purposes in order to provide additional guidance towards the location of interest (Figure 1C) [42,43].

#### 3.2.1. Gamma Probes

Use of gamma probes in robotic surgery has required adaptation of the gamma cameras and probes already used in open surgery [3,10,14,39]. This has resulted in the development and subsequent use of laparoscopic gamma probes. The first reports of the use of a laparoscopic gamma probe were by Jeschke et al., and later that year by Meinhardt et al. [44,45]. In these studies, a laparoscopic gamma probe was used for laparoscopic SLNB procedure of the prostate. Here, the majority of tumor-positive SNs were located around the bifurcation of the external and internal iliac artery. In later studies where a robot assisted (RA) SLNB was performed, this laparoscopic gamma probe was used, but here the robot arms limited the reach of the static laparoscopic gamma probe [14].

**Figure 2:**
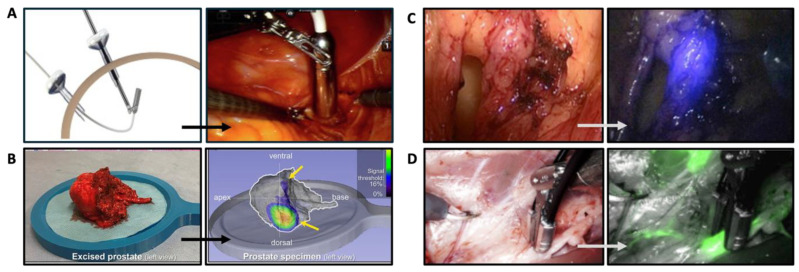
Devices for intraoperative guidance. (**A**) Drop-in gamma probe (Ref. [24]), (**B**) robotic SPECT (Ref. [49]), (**C**) fluorescence imaging (Ref. [14]), and (**D**) Click-on fluorescence probe (Ref. [50]).

Van Oosterom et al. demonstrated the limitations of the laparoscopic gamma probe, with the main drawback being the limited angles of detection, which ultimately leads to sub-optimal detection of the gamma signal [46]. To overcome this, a tethered miniaturized gamma probe (called the drop-in gamma probe) was designed to align the surgical need for gamma tracing with the dexterity improvements achieved by surgical robots [47,48]. This drop-in probe can be inserted via one of the trocars, and when located in the abdominal cavity, the surgeon is able to pick up and manipulate the detector with the steerable robotic instruments (Figure 2A). De Barros et al. reported the first analysis of PSMA-targeted radioguided surgery (RGS) using the drop-in gamma probe in patients with lymph node (LN) recurrent prostate cancer, with a sensitivity of 86% and a specificity of 100% [21]. In an ex vivo setting, it was also shown to be feasible to create a 3D SPECT image using this probe on prostatectomy samples, showing tracer uptake within the specimen [49]. Here, light detection and ranging (LiDAR) was used to provide anatomical reference, similar to CT imaging (Figure 2B). In vivo translation of this robotic SPECT approach is currently being performed.

Building on the initial drop-in probe results, a drop-in beta probe for consecutive use with beta tracers was also evaluated [24]. This probe was used to trace PSMA-expressing LNs in 7 primary prostate cancer patients. In this study, a signal-to-background ratio discrimination algorithm was used to discriminate between the signal of health and malignant tissue, rather than solely being guided by the acoustic signal and count rate by the probe.

#### 3.2.2. Fluorescence Laparoscopes

The first report of the use of a fluorescence laparoscope in RA SN detection for prostate cancer involved the introduction of an additional laparoscope [5]. In this approach, the light of the robot’s laparoscope was switched off, and the additional laparoscope (STORZ laparoscope) equipped with fluorescence imaging was deployed. This method allowed for the visualization of the ICG fluorescence signal, which resulted in enhanced detection accuracy of SNs during the procedure (Figure 2C, [14,51]). Further development of this approach was achieved after integration of the fluorescence laparoscope (Firefly, Intuitive) in the robotic system, which allows direct control by the urologist [3].

With the introduction of new generation robots such as Mazor X Stealth Edition, Medtronic (2018), Senhance Surgical System, TransEnterix (2017), Versius Surgical System, CMR Surgical (2019), and SPORT Surgical System, Titan Medical (2020) has expanded the range of systems available for fluorescence imaging in a robotic surgical setting [52]. Incorporating a fluorescence laparoscope directly into robotic surgical systems has the potential to simplify the process of fluorescence detection, particularly in procedures such as the SN procedure or tumor identification in hybrid surgical approaches [3]. Here it should be noted that to date all these ingrate endoscopes are tailored towards a use in combination with ICG (a Cy7.5 analogue), a feature that already limits their compatibility with Cy7 analogues and fluorescent dyes with a lower emission wavelength, such as Fluorescein or Cy5. At the same time, multispectral imaging has been put forward as a tool to simultaneously visualize multiple surgical (non-) targets based on discrimination between different fluorescent emissions [53].

Similar to rigid gamma probes, fluorescence laparoscopes are limited in their intraoperative movability. Using an analogous design strategy to that of the drop-in gamma probe, more dexterous fluorescence detection is currently being explored [50].

### 3.3. Navigation Techniques

#### 3.3.1. Electromagnetic Tracking

Electromagnetic tracking in navigation surgery involves the use of electromagnetic fields to track the position of surgical instruments in real-time. This technology allows for precise and accurate guidance during complex surgical procedures [48]. By tracking the instruments’ location relative to the patient’s anatomy, surgeons can ensure precise placement and minimize risk. In an SN procedure for prostate cancer patients, navigation with electromagnetic tracking of the surgical tool was evaluated by Aguilera Saiz et al. In this feasibility study 91% of the preoperatively defined SNs could be located with navigation-based electromagnetic tracking [54,55]. Here, the localization of the SN could be verified with the gamma and fluorescent ICG signal of ICG-^99m^Tc-nanocolloid.

#### 3.3.2. Near Infra-Red Optical Tracking

Near infrared (NIR) optical tracking during surgery involves using infrared light to track the position of surgical tools and instruments in real-time. This technology allows for precise navigation and guidance during surgical procedures [56]. By detecting the infrared markers on the instruments, the system can accurately track their location and movement within the surgical field. This enhances the accuracy and efficiency of the surgery, ultimately improving patient outcomes.

In 2018, a phantom and in vivo study by Oosterom et al. showed the feasibility of navigation of surgical tools (for example, a fluorescence laparoscope) towards the target of interest based on SPECT imaging overlay which was realized with near infrared optical tracking (Figure 2D) [43].

### 3.4. Clinical Indications

#### 3.4.1. Prostate Cancer

For prostate cancer, three RA surgical procedures are performed with hybrid surgical guidance, namely margin assessment during prostatectomy (overview of hybrid tracers in Table 1), the SLNB procedure (Table 2), and salvage procedures for identification of LN containing (macro-)metastatic disease in recurrent prostate cancer [12,13,14,39]. To date, the detection of metastatic disease is the most investigated application. In a systematic review, Berrens et al. [12] showed that the clinical implementation of ^99m^Tc-PSMA-RGS has improved the detection of positive LN metastases (>2 mm) compared to traditional extended pelvic lymph dissection (ePLND). However, the limitations of ^99m^Tc-PSMA agents include their inability to detect micro-LN metastasis (<2 mm) and lesions with low to intermediate levels of PSMA. A study by Hinsenfeld et al. revealed upstaging to pN1 in 26% of PSMA-PET negative patients after hybrid SLNB [57].

#### 3.4.2. Sentinel Lymph Node Biopsy

Two different hybrid tracer designs for use in SLNB procedures in prostate cancer have been described (Table 2). The most investigated approach is based on the hybrid SN tracer ICG-^99m^Tc-nancolloid. While not included in the treatment guidelines as first-line treatment in prostate cancer, the procedure was shown to be a reliable alternative for ePLND for staging prostate cancer patients, with a sensitivity of 95% [62]. Use of hybrid SLNB as a supplement to standard ePLND was associated with lower rates of biochemical recurrence (0.79; 95%CI, 0.63–0.98) and of clinical recurrence (hazard ratio, 0.76, *p* = 0.035) compared to ePLND treatment only [40]. In some medical centers in the Netherlands, the SLNB procedure is standard of care as a replacement for ePLND for staging in patients who are scheduled for external beam therapy [63]. This approach aims to eliminate the need for an intensive exploration of all non-significant LN with ePLND, with the aim of reducing complication rates and the potential risk of damage to vital structures [64].

Recently, an MRI-based hybrid tracer was introduced, with a focus on the use of SPION for magnetic resonance imaging (MRI) and handheld magnetometer probe detection in combination with intraoperative fluorescence identification based on ICG [60]. This approach allows anatomical MRI to serve as a surgical guidance plan for intraoperative fluorescence imaging in a consecutive, but not directly combined, manner.

#### 3.4.3. PSMA Targeted Surgery

**Figure 3:**
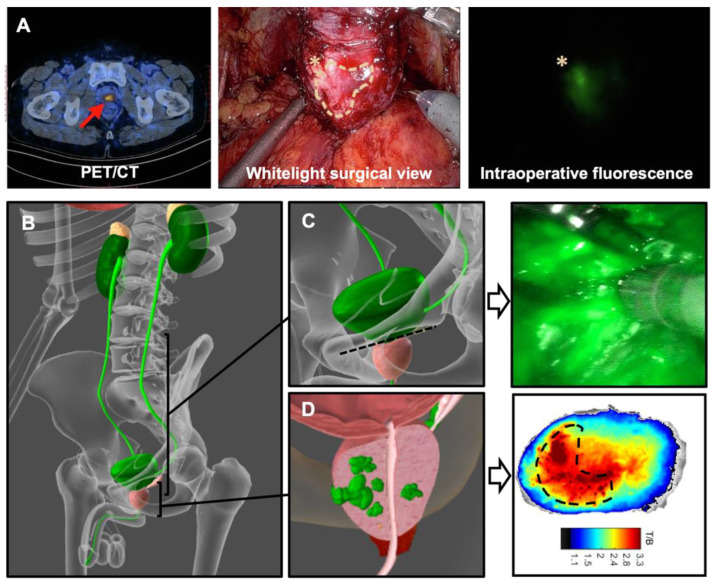
Tracer clearance and tumour visualization. (**A**) Clinical example: the use of the hy-brid PSMA-targeting tracer 68Ga-P3 in robotic surgery (Red arrow: tumor location on PSMA-PET/CT; * : reference point for white light and fluorescence imaging; white dashed stripe: tumor location prostate, (adapted from ref. [66])). (**B**) schematic rep-resentation of the renal clearance pathway. (**C**) Dislocation prostate from tracer-containing bladder during prostatectomy (**left** image) and contamination with fluorescent dye-containing urine in the surgical field after prostatectomy in a prostate cancer patient (**right** image; images obtained from ref. [37]). (**D**) Prostate cancer (**left** image) and fluorescence-based discrimination between lesions and surrounding tissue (**right** image; fluorescence image obtained from ref. [67], dashed = location tu-mor). Anatomical images created using Biodigital.com.

In the literature use of hybrid PSMA-targeting tracers (Table 1) still remains limited to first-in-human feasibility studies. Three separate studies have evaluated hybrid tracers based on ^68^Ga so suitable for preoperative imaging with PET/CT, each containing a different fluorescent component [57,58,59]. In a case study in a high-risk prostate carcinoma (Gleason score 9 (4 + 5), initial prostate specific antigen (PSA) level 7 ng/mL), Eder et al. [61] showed results of preoperative PET/CT imaging at one hour after intravenous administration of ^68^Ga-Glu-urea-Lys-(HE)_3_-HBED-CC-IRDye800CW (a derivative of ^68^Ga-PSMA-914) and subsequent RA fluorescence imaging (DaVinci Firefly camera) revealing strong tracer uptake of the primary tumor located in the left prostate lobe. Chen et al. evaluated ^68^Ga-P3, a hybrid tracer containing an ODAP-Urea-based PSMA-targeting moiety, a radiometal chelator, and an ICG analog, in 16 prostate cancer patients under three different dosing regimens (10 µg/kg, 20 µg/kg, and 40 µg/kg) [58]. PET/CT imaging was performed at 30, 60, and 120 min after injection, and RARP with intraoperative fluorescence imaging was performed at 24 ± 6 h after injection. Imaging at 120 min post-injection revealed the highest TBR of the SUVmax and was shown to provide the optimal image contrast (Figure 3A). Although not evaluated for hybrid PSMA-targeting tracers yet in the clinical setting, optimization of dosing and timing of imaging is also an important feature for the utility of the fluorescent component. This was especially highlighted during intraoperative use of fluorescent tracers (e.g., OTL78 and IS-002, [65,66]) wherein fluorescence-based contamination of the surgical field, particularly after incision of the bladder neck (Figure 3B,C, [58]), was reported as a limitation and uptake in non-PSMA expressing tissue resulted in suboptimal TBR. In the preclinical setting Dell’Oglio et al. [67] showed a decreasing presence of the hybrid PSMA tracer ^99m^Tc-EuK-(SO(3))Cy5-mas3 (*h*PSMA) in urine over time, with an optimal timepoint of imaging (without fluorescent contamination of the surgical field) similar to that of PSMA-I&S. This tracer also allowed tumor visualization in ex vivo human prostate samples (Figure 3D).

In a different approach to standard fluorescence imaging, one study evaluated the hybrid surgical concept for RA prostatectomy using ^68^Ga-PSMA and CLI for visualization of the tumor within the prostate [9]. This approach allowed identification of the tumor itself, as well as detection of positive surgical margins. In Table 1, the different clinical available hybrid PSMA tracers are described; the sensitivity for tumor identification ranges from 64–100%.

### 3.5. Kidney Cancer

#### 3.5.1. Sentinel Lymph Node Biopsy

In a recent review by van Gennep et al. [68], various tracers for SN mapping in urology were described. However, this evaluation revealed that the current literature only describes the use of radioguided surgery for SN mapping during RA partial or radical nephrectomy [69] and that there are currently no reports available that describe the use of hybrid tracers for this indication. SLNB in renal cancer still lags well behind its penile and pelvic counterparts and has some way to go before widespread implementation can be considered. In addition, evaluation being limited to small study cohorts, there are concerns about procedural sensitivity (in particular, false negative outcomes) and patient selection criteria [68]. Mahesan et al. suggest that image-guidance and, in particular, the addition of fluorescence imaging could be a main contender in improving SLNB in kidney cancer [70]. This also suggests a role for hybrid SLNB tracers and image guidance approaches.

#### 3.5.2. Tumor Targeted Surgery

ICG is widely used as a fluorescent agent for vascular identification; it may also be valuable for tumor identification in partial nephrectomies [71,72]. Herein, different fluorescence patterns have been shown, namely a fluorescent (no visible uptake of dye), hypofluorescent (uptake of dye, but less than parenchyma), or isofluorescent (uptake of dye at intensity indistinguishable from surrounding parenchyma) [72]. Mass hypofluorescence with ICG NIRF was able to predict malignancy with a sensitivity of 84%, specificity of 57%, and positive predictive value of 87% and negative predictive value of 52% (Table 3). OTL38 [73,74], a fluorescent targeted probe, was analyzed for tumor visualization within the kidney (Table 3). In an ex vivo perfusion study, the potential of the hybrid carbonic anhydrase IX (CAIX) targeting antibody-based tracer ^111^In-girentuximab-IRDye800CW was shown for visualization of the tumor within the kidney with a high sensitivity [41].

### 3.6. Bladder Cancer

#### 3.6.1. Sentinel Lymph Node Biopsy

**Figure 4:**
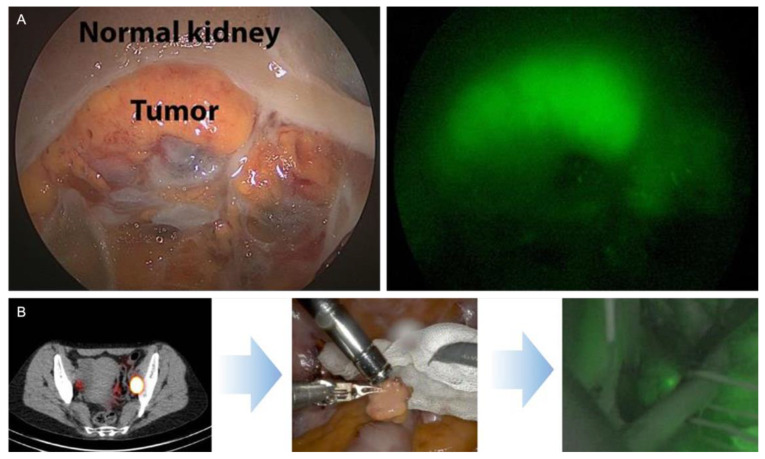
Hybrid image guidance in renal and kidney cancer. (**A**) Fluorescence-guided tumor visualization using the hybrid carbonic anhydrase IX (CAIX) targeting antibody-based tracer ^111^In-girentuximab-IRDye800CW in an ex vivo kidney perfusion model [41]. (**B**) Hybrid sentinel node imaging based on combined pre- and intraoperative guidance using the hybrid tracer ICG-^99m^Tc-nanocolloid [16,75].

In bladder cancer, only 2 studies describe a hybrid surgical guidance concept for SLNB during open and RA cystectomy [16,75] (Table 4, Figure 4B). In these studies, the SN of the bladder was evaluated using ICG-^99m^Tc-nanocolloid. Rietbergen et al. [73] showed that the location of the tumor and, as such, the injection site was important for where to find the SN in the pelvic area. Intraoperative findings were limited to the location of the SNs (80% of SNs were found within the ePLND template, 53% in the obturator fossa, and 27% at the external iliac artery) [75], and these locations were in agreement with the locations identified on preoperative imaging.

In a study by van Gennep et al. [16], a sensitivity of 85.7% and a false negative rate of 14.3% were reported, which is in line with values in literature obtained with the gold standard ^99m^Tc-based albumins [76,77,78]. However, a relatively high non-visualization rate of 36.7% was found, and survival analysis showed poor survival for the patients with a non-visualization and a positive LN in the PLND [16]. As such, this study concluded that in the case of non-visualization, a PLND should be performed.

#### 3.6.2. Tumor Targeted Tracers

For bladder cancer, there are currently no tumor-targeted agents described that are suitable for (hybrid] radio- or fluorescence-guided surgery. For bladder cancer, a possible compound for fluorescence-guided surgery is evaluated in a preclinical setting, namely MNPR-101-800F for urokinase plasminogen activator receptor (uPAR) fluorescence imaging [79]. To our knowledge, there are no clinical hybrid tracer derivatives available for the surgical approach for bladder cancer.

## 4. Discussion

The field of urologic robotic oncological surgery has seen advancements in surgical techniques, including the use of hybrid tracers for non-invasive diagnostics and supplementary intraoperative imaging. The latter is supported by the increasing development and clinical application of devices, such as (drop-in) gamma or beta probes and fluorescence laparoscopes, that provide additional guidance to aid in intraoperative detection of SNs or tumors.

Fluorescent tracers like ICG are commonly used in uro-oncology, especially for RA (partial) nephrectomies (Table 3). Clearly, the detection rates of ICG alone leave ample room for improvement provided by hybrid targeted tracers. This assumption is further substantiated by results obtained with the folate-targeting fluorescent tracer OTL38 [73,74]. However, hybrid tumor targeted agents are not yet used in the clinical setting in robot assisted (partial) nephrectomies. The hybrid tracer ^111^In-girentuximab-IRDye800CW could potentially be a good candidate for hybrid surgery in procedures such as RA partial nephrectomy(Figure 4A, [41]).

Hybrid tracers combine the preoperative imaging capabilities of radiotracers with the possibility to visually confirm localization and excision of the tissue of interest [11]. Moreover, preoperative imaging provides more detailed three-dimensional information that has been shown to be crucial for effective excision of the SN [80]. This information can also include details about the tumor’s depth and its relationship with possible surrounding structures [48,55]. In addition, hybrid tracers make use of the complementary features of both imaging approaches to minimize the effect of individual technological weaknesses during intraoperative molecular image guidance. Herein, the excellent tissue penetration of gamma and beta radio signals provides a means to roughly guide the surgeon towards the SN or tumor, while the limited signal penetration of several millimeters of fluorescence imaging enables high-definition and real-time confirmation during resection. Together, such hybrid imaging allows for identification of both superficial and more endophytic lesions and even allows identification of SNs located in close proximity to the (high signal intensity) injection site [11]. Tracer development has been duly followed by clinical application of novel devices that enable more accurate intraoperative guidance and detection. Herein creation of unique technological designs, such as drop-in probes that can be handled by the surgeon using the surgical tools [16,21,24,47] and integration of fluorescence laparoscopes in the surgical robot [32] have enabled compatibility with the robotic setting. The drop-in (gamma or beta) probe was evaluated in prostate cancer surgery, but with a possible localization of SN or tumor-positive LN in the pelvic region, it can also be used for bladder cancer surgery.

Evaluation of most of the novel intraoperative devices and technologies was first performed in SLNB [36]. The success of the hybrid tracer ICG-^99m^Tc-nanocolloid in SLNB underlines the contribution of harmonization of the different biodistribution profiles between tracer components and subsequent elimination of discrepancies between preoperative and intraoperative imaging results [3,10], as this tracer was shown to outperform single modality-based methodologies based on the use of blue dye and free ICG [10,39]. Together with obtained improvements in biochemical recurrence (0.79; 95%CI, 0.63–0.98) and of clinical recurrence (hazard ratio, 0.76, *p* = 0.035) for hybrid SLNB compared to ePLND treatment in prostate cancer [40] the additional guidance provided by this tracer provides opportunities for application and/or improvement of SLNB in less investigated tumor types such as bladder and kidney cancer. In bladder cancer, the use of a hybrid tracer for SLNB has shown promising results, with an acceptable sensitivity- and low false-negative rate [16]. These findings underscore the potential of hybrid tracers in improving the staging of LN in bladder cancer. This could particularly be relevant in guiding decision-making and reducing the need for ePLND in the future. While LN status is a major prognostic indicator in kidney cancer, SNLB still remains experimental, with suboptimal detection rates and false-negative rates being the main limitations for its clinical usefulness [68]. As applied in other tumor types, improvement of the injection technique [81] may contribute to higher performance rates. As age and a higher body mass index have also been attributed to playing a role in non-visualization [68,82], more conservative patient selection might also result in improved utility of (hybrid) SLNB in kidney cancer.

Currently, the most investigated example of tumor-specific molecular imaging approaches is PSMA-targeted surgery using the radiotracer ^99m^Tc-PSMA-I&S in prostate cancer [18], a chemical design that has been followed by various hybrid analogues [12]. For targeted imaging, the small peptides (e.g., in PSMA-targeted tracers [35,83]) are often preferred over the use of antibodies. This is due to their more optimal pharmacological characteristics that result in a logistically more favorable time window between tracer administration and surgery [35]. From a chemical perspective, however, reduction of the molecular size complicates the tracer design. Hereby, the wrong dye in the wrong location can harm the receptor affinity and/or mess up the pharmacokinetics. For PSMA, for example, this means multiple iterations are often required to come up with the optimal tracer [84]. As different tumor types show overexpression of different receptors (e.g., CAIX or UPAR [41,79]), this calls for the development and optimization of tracer variants per individual receptor and subsequent receptor-pattern dependent tracer selection per tumor, (type) [35]. To date, evaluation of tumor-specific intraoperative tumor and margin detection in bladder and kidney cancer remains limited to a few (ex vivo) examples [41,79], Still in the results obtained with PSMA indicating a promising avenue for exploring tumor targeted hybrid tracers in future research and clinical applications in bladder and kidney cancer as well, particularly with the aim to visualization of tumor margins and aid in tumor identification in partial nephrectomies.

Besides selection, of the most appropriate hybrid tracer, dosing and timing of imaging also play a prominent role in the possible success of hybrid tumor-specific imaging. Based on expert consensus, the optimal timing for diagnostic PSMA-ligand PET/CT before surgery was deemed to be within 1 to 3 months [19]. The optimal interval between injection of the PSMA tracer and intraoperative imaging should not exceed 16–20 h, which is the general time window applied for PSMA-targeted radiotracers [12,18,85]. In the preclinical setting, Dell’Oglio et al. showed the feasibility of visualization of PSMA expression using ^99m^Tc-*h*PSMA in the porcine prostate within this timeframe at a microdosing regimen similar to that applied for radiotracers [67]. Clinical translation of this hybrid tracer is currently ongoing). Pharmacokinetic evaluation has shown a decrease of fluorescence in urine and other tissues surrounding the prostate [58,67,85]. This is also thought to decrease fluorescence-based contamination of the surgical field, particularly after incision of the bladder neck (Figure 3B,C, [35]) and improve tumor visualization (Figure 3D). In the current clinical such contamination has been reported to hinder intraoperative assessment of the basal margin of the prostate as seen with the fluorescent PSMA-targeting tracer OTL78 [66], but this also applies to visualization of fluorescence-containing lymph nodes located close to the prostate. Based on experience with fluorescent PSMA-targeting tracers (generally applied under therapeutic dosing (mg/kg), [65,66]), it has become clear that dosing can have a profound effect on tumor visualization and false-positive findings. In vivo fluorescence imaging under a microdosing regimen (100 ug/patient) has been shown to be feasible in the preclinical setting [67] but still needs to be confirmed in the clinical setting. Here, it is important to note that signal-to-background ratios for targeted approaches have been shown to be significantly lower compared to ratios obtained for SLNB [86].

The importance of utilizing both SLNB and PSMA targeted approaches in clinical practice was highlighted in a study conducted by Hinsenveld et al. [57] The study demonstrated that 26% of PSMA-PET negative patients were upstaged to pN1 after undergoing hybrid SLNB, emphasizing the critical need for both techniques. These findings underscore the significance of incorporating both SLNB and targeted approaches and suggest a promising future for hybrid PSMA-targeted guidance.

This narrative review reveals that the majority of advancements in hybrid surgery have been made in RA prostate cancer surgery. This presents an opportunity to shift focus towards the development of appropriate hybrid tracers for kidney and bladder cancer, as well as to expand upon the techniques already established for prostate cancer.

## 5. Future Directions

Through the many globally ongoing (radio)chemical efforts, the pool of hybrid tracers suitable for a wide range of targets that can potentially be translated to the clinic is rapidly growing. Thereby opening the way for multiple Robotic-urology indications to benefit from the hybrid concept. Translation that will be catalyzed when the chemical designs are not only GMP-compatible and non-toxic, but also align with the unmet clinical needs and are compatible with available clinical modalities. That said, we have seen that the clinical introduction of hybrid SLNB in 2009 has promoted a range of technical innovations, such as first-in-robot near-infrared fluorescence laparoscopy [5], navigation to fluorescence targets [43], hybrid imaging modalities [36], and the clinical use of a drop-in gamma probe [87]. It is, therefore, to be expected that expansion of clinical trials that use a hybrid tracer will also boost future engineering efforts. When chemical and engineering innovations are synchronized with each other and fully refined, the expectation is that these will help achieve improved outcomes throughout the field. This will, however, require more prospective clinical evaluations that also establish the clinical utility of these tracers and guide their widespread adoption in surgical practice.

## 6. Conclusions

The use of hybrid tracers and matching imaging devices seems to improve detection rates and guidance during robotic procedures for prostate, bladder, and kidney cancer. Where the hybrid tracers can also be used in an open surgery setting, the robotic setting tends to require tailored imaging devices for them to create optimal impact in either primary or salvage surgery. For kidney cancer and bladder cancer, developments are lagging in relation to prostate cancer. Future research should focus on developing new strategies to enhance imaging accuracy and expand the use of hybrid tracers (tumor targeted) in RA uro-oncological surgical procedures.

## Figures and Tables

**Table 1:** Hybrid targeted agents for introperative **clinical use** in (robot assisted) prostatectomy.

Reference	N	Surgical Procedure	Tracer	Preoperative Imaging	Intraoperative Imaging	Detection Rate
Darr et al. [9]	10	Prostatectomy (not RA)	^68^Ga-PSMA	PET/CT	Cerenkov luminescence imaging LightPath CLI system (Lightpoint Medical Ltd.).	3 positive surgical margins/2 detected with CLI Sensitivity 66%
Chen et al. [58]	16	RA prostatectomy	^68^Ga-P3	PET/CT	Firefly fluorescence imaging (Intuitive Surgical)	Sensitivity 79.1%, specificity 90.4%, PPV 81.5%, NPV 89.0%
Eder AC et al. [59]	1	RA prostatectomy	PSMA-11-derived hybrid molecule PSMA-914 (^68^Ga-Glu-ureaLys-(HE)3-HBED-CC-IRDye800CW)	PET/CT	Firefly fluorescence imaging (Intuitive Surgical)	NA
Aras et al. [60]	10	RA prostatectomy	[^18^F]-BF3-Cy3-ACUPA	PET/CT 6 patient PET/CT/MRI	Custom-made fluorescence imager	NA

RA = robot assisted, ePLND = extended pelvic lymph node dissection, ^68^Ga = Gallium- 68, PET/CT = Positron emission tomography/Computed tomography, CLI = Cerenkov luminensence imaging, NA = not answered, NIR = Near infrared imaging, PPV = positive predictive value, NPV = negative predictive value, MRI = Magnetic Resonance Imaging.

**Table 2:** Examples of robot assisted hybrid sentinel node in **clinical** studies in prostate cancer.

Sentinel Node Studies	N	Surgical Procedure	Tracer	Hybrid Yes/No	Preoperative Imaging	Intraoperative Imaging	Detection Rate
Michalik B et al. [61]	10	Prostatectomy + ePLND No robot surgery	Superparamagnetic iron oxide nanoparticles (SPION) and indocyanine green	Yes	MRI	NIR optical imaging system (QUEST SPECTRUM 3, Olympus, Hamburg, Germany) Handheld magnetometer probe (Sentimag, Endomag, Cambridge, UK)	70% concordance for preoperative MRI vs. magnetometer-guided PLND 88% concordance for magnetic vs. fluorescent SLN detection. Sensitivity and specificity NA
Wit EMK et al. [10]	138	RA prostatectomy + SLNB	ICG-^99m^Tc-nancolloid Vs. ^99m^Tc-nancolloid + “free ICG”	Yes	SPECT/CT	Firefly fluorescence imaging, Intuitive Surgical	NA
KleinJan GH et al. [3]	55	RA prostatectomy + SLNB	ICG-^99m^Tc-nancolloid	Yes	SPECT/CT	Firefly fluorescence imaging, Intuitive Surgical	Sensitivity 92.9% FNR 7.1%
KleinJan GH et al. [14]	40	RA prostatectomy + SLNB	ICG-^99m^Tc-nancolloid	Yes	SPECT/CT	Karl Storz laparoscopes +lap gamma probe	Sensitivity 75% FNR 14%
Van der Poel et al. [5]	11	RA prostatectomy + SLNB	ICG-^99m^Tc-nancolloid	Yes	SPECT/CT	Karl Storz laparoscopes + lap gamma probe	NA

RA = robot assisted, ePLND = extended pelvic lymph node dissection, SLNB = sentinel lymph node dissection, ICG- (^99m^Tc)-nanocolloid = Indocyanine Green-Technetium 99m-nanocolloid, SPECT/CT = Single photon emission tomography/Computed tomography, MRI = Magnetic Resonance Imaging, NA = not answered, NIR = Near infrared imaging, PPV = positive predictive value, NPV = negative predictive value, FNR = False negative rate.

**Table 3:** Examples of fluorescent and hybrid imaging agents used in **clinical setting** in urological robotic surgery for partial nephrectomy.

Reference	N	Surgical Procedure	Tracer	Hybrid Yes/No	Preoperative Imaging	Intraoperative Imaging	Detection Rate
Hekman MC et al. [41]	8	Open and laparoscopic nephrectomy	Indium-111-DOTA-gerentuximab-IRDye800CW	Yes	SPECT 111-Indium	Fluorescence laparoscope Storz D-light P	Sensitivity 100%
Sulek et al. [74]	10	RA Partial nephrectomy	OTL38	No	NA	Firefly fluorescence imaging, Intuitive Surgical	Safety and effectiveness study Negative contrast
Tobis et al. [71]	19	RA Partial nephrectomy	ICG	No	NA	Endoscopic SPY Imaging System	NA 11 patients 7 hypo-fluorescent 3 iso fluorescent
Manny et al. [72]	100	RA Partial nephrectomy	ICG	No	NA	Firefly fluorescence imaging, Intuitive Surgical	Sensitivity 84% Specificity 57%. PPV 87% NPV 52%,

RA = robot assisted, ICG = Indocyanine Green, SPECT = Single photon emission tomography, NA = not answered, PPV = positive predictive value, NPV = negative predictive value.

**Table 4:** Hybrid imaging agents used in **clinical setting** in urological robotic surgery for bladder cancer.

Reference	N	Surgical Procedure	Tracer	Hybrid Yes/No	Preoperative Imaging	Intraoperative Imaging	Detection Rate
Rietbergen et al. [75]	20	Open and RA cystectomy SLNB	ICG-^99m^Tc-nanocolloid	Yes	SPECT/CT	Gamma probe Firefly fluorescence imaging, Intuitive Surgical	SPECT/CT 53% Sensitivity NA
Van Gennep et al. [16]	30	Open and RA cystectomy SLNB	ICG-^99m^Tc-nanocolloid	Yes	SPECT/CT	Gamma probe + drop-in gamma probe Firefly fluorescence imaging, Intuitive Surgical	Sensitivity 85.7% FNR14.3%

RA = robot assisted, ePLND = extended pelvic lymph node dissection, SLNB = sentinel lymph node dissection, ICG- (^99m^Tc)-nanocolloid = Indocyanine Green-Technetium 99m-nanocolloid, SPECT/CT = Single photon emission tomography/Computed tomography, NA = not answered, FNR = false negative rate.

## Abbreviations

The following abbreviations are used in this manuscript:

SLNB	sentinel lymph node biopsy
SN	sentinel node
RA	Robot-assisted
ICG	indocyanine green
PSMA	prostate specific membrane antigen
PET	positron emission tomography
SPECT	single photon emission computed tomography
CT	computed tomography
CAIX	carbonic anhydrase IX
CLI	Cerenkov luminescence imaging
NIR	Near infrared
^68^Ga	Gallium-68
^99m^Tc	Technetium-99m
^111^ In	Indium-111
uPAR	urokinase plasminogen activator receptor
LN	lymph node
ePLND	extended pelvic lymph node dissection
